# EVALUATING THE IMPLEMENTATION OF A SELF-CARE INTERVENTION FOR CAREGIVERS OF PERSONS WITH DEMENTIA

**DOI:** 10.1093/geroni/igad104.2750

**Published:** 2023-12-21

**Authors:** Lauren Fisher, Lauren Massimo, Karen Hirschman

**Affiliations:** University of Pennsylvania, Philadelphia, Pennsylvania, United States; University of Pennsylvania, Philadelphia, Pennsylvania, United States; University of Pennsylvania, Philadelphia, Pennsylvania, United States

## Abstract

In this study, we evaluated the implementation of the Virtual Caregiver Coach for You (ViCCY) intervention delivered as part of the “iCare4Me for FTD” (Frontotemporal Degeneration) Study (NCT04686266). This randomized controlled trial was designed to evaluate the efficacy of the evidence-based ViCCY intervention designed to increase self-care in caregivers of persons living with FTD. All caregivers received health information (HI) which included targeted caregiver and FTD websites on an iPad. The intervention group received ViCCY, 10 health coaching sessions focused on self-care and delivered via an iPad over 6 months. Guided by the Consolidated Framework for Intervention Fidelity (CFIF), the goal of this sub-study was to evaluate intervention implementation by measuring key CFIF components: coverage, exposure, and content delivered. Thirty-one caregivers were randomly assigned to ViCCY+HI (n=15) or HI only (n=16). One caregiver withdrew from the HI group. For exposure, at Month 1, a higher number of HI participants (87%) reported reviewing the websites 30 minutes a week compared to ViCCY group (67%). By Months 3 and 6 the groups were identical (3M: 100%, 6M: 93%). Thirteen out of 15 ViCCY caregivers completed all 10 health coaching sessions. On average, these sessions lasted 52 minutes (range: 24.8-113.5). For content adherence, 28% (40/142) of the completed sessions were examined for adherence to the protocol across the 10 sessions. Overall, content adherence across all sessions was 96% (range: 92-100%). This evaluation demonstrated that a virtual intervention targeting self-care is feasible and acceptable in this population. Implications for future research will be discussed.

